# Antifungal Activity of ZnO Nanoparticles Synthesized from *Eichhornia crassipes* Extract for Construction Applications

**DOI:** 10.3390/nano14121007

**Published:** 2024-06-11

**Authors:** Rocío Vargas Hernández, Mayra A. Alvarez Lemus, Susana De la Rosa García, Rosendo López González, Patricia Quintana, David García Zaleta, Viridiana Velázquez Vázquez, Sergio Gómez Cornelio

**Affiliations:** 1 Multidisciplinary Academic Division of Jalpa de Méndez, Juarez Autonomous University of Tabasco, Jalpa de Méndez 86205, Tabasco, Mexicodavid.garcia@ujat.mx (D.G.Z.); 2 Nanotechnology Laboratory, Academic Division of Engineering and Architecture, Juarez Autonomous University of Tabasco, Cunduacán 86690, Tabasco, Mexico; mayra.alvarez@ujat.mx (M.A.A.L.);; 3 Laboratory of Applied Microbiology, Academic Division of Biological Sciences, Juarez Autonomous University of Tabasco, Villahermosa 86000, Tabasco, Mexico; 4 National Laboratory of Nano and Biomaterials, Department of Applied Physics, CINVESTAV-IPN, Cordemex, P.O. Box 73, Mérida 97310, Yucatán, Mexico; 5 Biotechnology Laboratory, Polytechnic University of the Center, Centro 86290, Tabasco, Mexico

**Keywords:** sick building syndrome, fungicidal properties, bio-assisted synthesis, MIC/MFC ratios, construction materials

## Abstract

Fungal growth on construction materials in tropical climates can degrade aesthetics and manifestations on modern and historical sick buildings, affecting the health of their inhabitants. This study synthesized ZnO nanoparticles with enhanced antifungal properties using a precipitation method. Different concentrations (25%, 50%, and 100%) of *Eichhornia crassipes* aqueous extract were used with Zn(NO_3_)_2_·6H_2_O as the precursor to evaluate their spectroscopic, morphological, textural, and antifungal properties. X-ray diffraction confirmed the hexagonal wurtzite phase of ZnO with crystallite sizes up to 20 nm. Fourier-transform infrared spectroscopy identified absorption bands at 426, 503, and 567 cm^−1^ for ZnO-100, ZnO-50, and ZnO-25, respectively. Nitrogen physisorption indicated a type II isotherm with macropores and a fractal dimension coefficient near 2 across all concentrations. Polydispersity index analysis showed that ZnO-50 had a higher PDI, indicating a broader size distribution, while ZnO-25 and ZnO-100 exhibited lower PDI values, reflecting uniform and monodisperse particle sizes. FESEM observations revealed semi-spherical ZnO morphologies prone to agglomeration, particularly in ZnO-25. Antifungal tests highlighted ZnO-25 as the most effective, especially against *Phoma* sp. with an MFC/MIC ratio of 78 µg/mL. Poisoned plate assays demonstrated over 50% inhibition at 312 µg/mL for all tested fungi, outperforming commercial antifungals. The results indicate that ZnO NPs synthesized using *E. crassipes* extract effectively inhibit fungal growth on construction materials. This procedure offers a practical approach to improving the durability of building aesthetics and may contribute to reducing the health risks associated with exposure to fungal compounds.

## 1. Introduction

In tropical climates, the continuous deterioration of both historical and modern structures due to the metabolic activity of microorganisms such as fungi poses a significant challenge [1]. These organisms release allergenic compounds and secondary metabolites that negatively impact human health, increasing the risks of respiratory diseases and contributing to sick building syndrome (SBS) [2,3]. Nanotechnology offers promising alternatives to address these challenges. It has been recently reported that *Zinc Oxide (ZnO) Nanoparticles (NPs)* may have dual activity as photocatalysts and antimicrobial agents [2,4], which has attracted the attention of researchers seeking to develop new alternatives to protect exposed surfaces, such as buildings.

Recent studies have investigated the synthesis of metallic NPs using plant extracts as an alternative to traditional chemical agents for reducing metal ions [5]. Additionally, these extracts act as stabilizing agents in the synthesis of metal oxides, including ZnO, where metabolites such as polyphenols and flavonoids reduce reactive oxygen species, offering a more sustainable and efficient synthesis method [6]. However, the diversity and variability of the active compounds present in the extracts propose challenges in determining the exact synthesis route of metal oxides [7].

Despite being considered an invasive species that destabilizes aquatic ecosystems by competing with native species and reducing the oxygen exchange necessary for aquatic life [8], *Eichhornia crassipes* has shown significant potential in nanotechnology. Studies have demonstrated that extracts from this plant are effective in the synthesis of ZnO NPs using various precursors. For instance, using zinc nitrate with the extract of this plant not only improves the optical and morphological properties of the NPs [9] but also, when using zinc acetate, enhances their antimicrobial activity [10,11]. This study focuses on evaluating the antifungal properties of ZnO NPs assisted by *E. crassipes* extract, analyzing different concentrations of the extract and their impact on the physical and chemical properties of ZnO. The main goal is to determine the optimal concentration of the extract that maximizes the antifungal effectiveness of the NPs for potential application in the construction sector.

## 2. Materials and Methods

### 2.1. Obtaining Eichhornia crassipes Extract

*Eichhornia crassipes* leaves were collected from the Negro Lagoon in Tabasco, Mexico. The leaves were rinsed with distilled water to remove any surface contaminants and then subjected to drying in a food dehydrator (Chr-20; Cuisinart, Stamford, CT, USA) for 5 h at a medium heat setting. The aqueous extract was prepared by adding 10 g of dried *E. crassipes* leaves to 200 mL of deionized water. The mixture was heated at 80 °C and agitated continuously for 10 min. Afterwards, the mixture was allowed to cool to room temperature and subsequently filtered by gravity to separate the solid biomass. To assess the impact of extract concentration on ZnO NPs synthesis, the concentrated extract was further diluted to 50% and 25%.

### 2.2. Synthesis of ZnO Nanoparticles

The synthesis of ZnO NPs was conducted according to the procedure described by López et al. [12], with minor modifications. For each synthesis, 100 mL of *E. crassipes* aqueous extract at three different concentrations (100, 50, and 25%) was treated with 7.5 g of Zn(NO_3_)_2_·6H_2_O (≥98%, Sigma-Aldrich, St Louis, MA, USA). The mixture was continuously agitated for 5 min. Subsequently, a solution containing 2.6 g of Na_2_CO_3_ (≥99%, Sigma-Aldrich, St Louis, MA, USA) in 100 mL of deionized water was added, with stirring for 2 h. Once the reaction finished, the precipitate was vacuum-filtered and washed with 500 mL of deionized water to remove residual reactants. The filtered material was then dried in an oven (Ecoshel 9023A, Leverett, MA, USA) at 70 °C for 18 h. The dried samples were calcined at 500 °C for 4 h with a temperature ramp of 2 °C/min (TE-M12DR muffle furnace, Terlab, México). The resultant NPs were labeled as ZnO-100 (concentrated extract), ZnO-50 (50% diluted extract), and ZnO-25 (25% diluted extract).

### 2.3. Characterization of ZnO Nanomaterials

ZnO NPs were characterized using X-ray diffraction (XRD) to determine their crystalline phase. Diffraction patterns were acquired with a Bruker Advance ECO D8 powder X-ray diffractometer (Billerica, MA, USA), equipped with a LynxEye detector and a Cu radiation source (λ = 1.5406 Å). The scanning range was set from 10° to 70° 2θ, with a step size of 0.02° and a time step of 0.4 s, 40 kV, and 25 mA. The crystallite sizes were calculated using the Scherrer equation (Equation (1)) taking the most intense peak as a reference.

(1) $$D=\frac{k\lambda }{\beta Cos\theta }$$

where *D* is the crystallite size, *k* is the Scherrer constant valued at 0.89, λ = 1.5406 Å, *β* is the full width at half maximum (FWHM) of the diffraction peak, and θ is the Bragg angle in radians.

The optical properties of the ZnO NPs were analyzed using a Shimadzu UV-2600 spectrophotometer (Kyoto, Japan), equipped with a diffuse reflectance integrating sphere, using BaSO_4_ (98% R) as the reference standard, across a wavelength range of 200 to 700 nm. The Kubelka–Munk method was applied to the spectral data to estimate the band gap energy (Eg). The specific surface area and surface texture of the ZnO NPs were characterized using a Quantachrome Autosorb 3B analyzer (Anton Paar QuantaTec, Inc., Boynton Beach, FL, USA). Nitrogen adsorption–desorption isotherms were recorded at 77 K following sample degassing at 300 °C. The results data were analyzed using AsiQwin software (V. 5.0, Antron Paar, Ashland, VA, USA).

Functional groups adhered to the ZnO NPs were analyzed using Fourier-transform infrared spectroscopy (FTIR) on a Nicolet iS50 Thermo Scientific spectrophotometer (Thermo Fisher Scientific, Waltham, MA, USA). Spectra were collected across the 4000–400 cm^−1^ range using 32 scans at a resolution setting. For the analysis, KBr pellets (99%; Meyer, Mexico) were prepared using a press (PIKE Technologies, Madison, WI, USA) at 3-ton pressure, containing 1% by weight of each ZnO sample. The hydrodynamic diameter and zeta potential of the ZnO NP samples were analyzed using a Zetasizer Nano ZS (Malvern Instruments, Ltd., Worcestershire, UK), equipped with a He-Ne laser operating at 632.89 nm and a detector positioned at 173°, according to manufacturer specifications. Solutions were prepared at a concentration of 0.01 mg/mL for analysis in Malvern DTS0012 and DTS1070 cells for dynamic light scattering (DLS) and zeta potential measurements, respectively. The samples were uniformly dispersed in sterile water. To determine the size and shape of the ZnO NPs, they were deposited onto carbon-coated 200-mesh copper grids and examined using field-emission scanning electron microscopy (FESEM, JSM-7601F, JEOL, Tokyo, Japan) operating at an acceleration voltage of 15 kV.

### 2.4. Antifungal Activity

Fungal strains were isolated from building walls exhibiting fungal growth using malt extract agar supplemented with 0.2% CaCO_3_ (MEAC). The morphological characterization of the fungi was performed by identifying their reproductive and vegetative structures, pigmentation, and the size and shape of conidia [13], according to the taxonomic keys established by Sutton [14] and Seifert et al. [15]. The purified strains were preserved in sterile saline solution (0.855%) and potato dextrose broth (PDB, BD Difco, Detroit, MI, USA) with 25% glycerol at −80 °C.

The antifungal activity of ZnO NPs was assessed using the broth microdilution method to determine the minimum inhibitory concentration (MIC), following a modified protocol from the Clinical and Laboratory Standards Institute [16]. Cultures of fungal strains aged 7–14 days were used to prepare a conidial suspension. The conidia were counted using a Neubauer chamber and adjusted to a concentration of 1 × 10^5^ conidia/mL in PDB. The assay was conducted in 96-well microplates with serial twofold dilutions, starting at 10,000 µg/mL in the first well and diluting to 78 µg/mL. Each well was inoculated with 100 µL of the fungal suspension. Benomyl at a concentration of 625 µg/mL served as the positive control for conidial germination inhibition, while the medium containing fungi without nanoparticles (NPs) was used as the negative control. The assays were conducted in quadruplicate, and the microplates were incubated for 24 h at 28 °C. The MIC was determined as the concentration at which conidial germination was not observed. As a confirmatory step, 30 µL of a 0.01% resazurin solution was added to each well.

Based on the obtained MIC values, a poisoned plate assay was conducted to determinate the antifungal activity of ZnO-25 NPs. The MEAC medium was prepared and sterilized, and at approximately 50 °C, ZnO-25 NPs were added at concentrations of 312, 624, and 1250 µg/mL. In the center of each plate, 8 mm discs with homogeneous fungal strain growth were inoculated. The plates were incubated at 28 °C, and the experiment was monitored until fungal growth on the control plates without NPs completely covered the Petri dishes. Itraconazole (6.6 mg/mL) was used as a chemical control. The assays were performed in triplicate. Mycelial growth inhibition (*MGI*) was calculated using the following formula [17]:

(2) $$MGI\;\left(\%\right)=\frac{dc-dct}{dc}\times 100$$

where *dc* represents the average diameter of fungal growth in the control without NPs, and *dct* represents the average diameter of fungal growth in the sample treated with NPs.

### 2.5. Statistical Analysis

The fungal growth data (in mm) from the poisoned plate assay were subjected to an analysis of variance (ANOVA) with a classification criterion, and the treatment means were compared using Tukey’s multiple comparison test. The homogeneity of variances and the normality of the data were verified using Levene’s test and the Shapiro–Wilk test, respectively. Statistical analyses were performed using PAST 3.0 software [18].

## 3. Results

**Figure 1:**
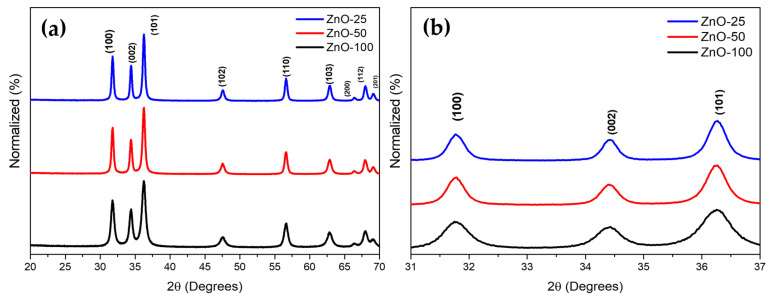
(**a**) X-ray diffraction patterns of ZnO NPs obtained from three different concentrations of *E. crassipes* extract, (**b**) detail for the 2θ = 31–37° interval.

The influence of extract concentration on the crystallinity of ZnO was analyzed using X-ray diffraction (XRD). The diffractogram (Figure 1) for the three ZnO NP samples shows the highest intensity peaks at the reflection planes (100), (002), and (101) located at 31.78°, 34.42°, and 36.26° (2θ), respectively (Figure 1b), consistent with the wurtzite-type ZnO structure (PDF # 00-036-1451) which is stable at temperatures below 900 °C. Variations in the peak width between ZnO-25 and ZnO-100 were observed. Furthermore, an increase in the concentration of the extract was found to correlate with smaller crystallite sizes, as calculated using the Scherrer equation (Table 1).

**Figure 2:**
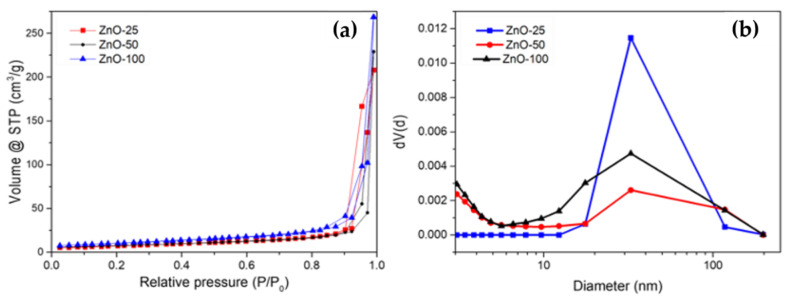
(**a**) Complete nitrogen adsorption–desorption isotherm for ZnO, and (**b**) pore size distribution of ZnO at different concentrations of *E. crassipes* extract calcined at 500 °C.

Diffuse reflectance spectroscopy (DRS) was used to estimate the band gaps (Eg) of ZnO NPs, which were calculated using the Kubelka–Munk method (Figure S1). The Eg values for ZnO varied slightly depending on the concentration, with a value of 3.22 eV for ZnO-50 and 3.27 eV for ZnO-100% (Table 1). Nitrogen physisorption was used to analyze the textural properties of ZnO NPs through nitrogen adsorption–desorption isotherms (Figure 2a) for the three concentrations of the extract. These isotherms were classified as type II according to the IUPAC classification.

The Brunauer–Emmett–Teller (BET) method was employed to calculate the specific surface area (Table 2) within the relative pressure range of 0.05 to 0.35. The obtained values for ZnO-100 were 36 m^2^/g and diminished to 26 m^2^/g for both samples with a lower concentration of *E. crassipes*, i.e., ZnO-25 and ZnO-50. However, the pore size values, calculated by the Barrett–Joyner–Halenda (BJH) method, for all ZnO NPs were very similar for diluted extracts (Figure 2b). Therefore, no difference was observed in the pore size distribution based on the adsorption isotherms. Additionally, the fractal dimension coefficient D (Table 2) was determined using the Frenkel–Halsey–Hill (FHH) model, which provides an estimation of the surface roughness; since for the three samples D was close to 2.3, a smooth surface can be assumed.

**Figure 3:**
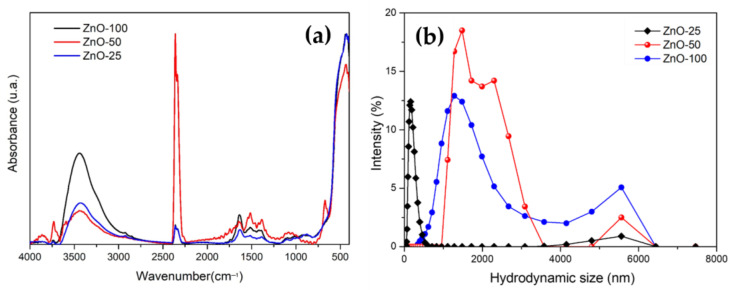
(**a**) Infrared spectrum of ZnO samples calcined at three concentrations of *E. crassipes* extract. (**b**) Hydrodynamic diameter distribution of ZnO from dynamic light scattering (DLS) measurements.

The infrared spectrum displays bands at 3400 cm^−1^ and 2800 cm^−1^, generally associated with O–H bond vibrations associated with physically adsorbed water from the ambient atmosphere, as well as functional groups from organic residues derived from the *E. crassipes* extract that were not completely degraded during the thermal treatment (Figure 3a). As can be seen, a strong and well-defined peak at 2360 cm^−1^ is present in all the samples, corresponding to the ambient CO_2_, as confirmed by the background of the spectra. Additionally, a small band around 670 cm^−1^ is observed, which corresponds to another vibration mode for CO_2_. The stretching vibrations of Zn-O from the hexagonal phase synthesized by chemical methods are found between 400 and 500 cm^−1^. For ZnO-100, a band is observed at 443 cm^−1^, while for the concentrations of ZnO-50 and ZnO-25, a shift to higher wavenumbers at 437 cm^−1^ is noted. A low-intense band located at 1736 cm^−1^ is also observed and can be assigned to C=O vibrations. This indicates that even after the thermal treatment, these functional groups are present, albeit with reduced intensity.

Given that ZnO NPs are suspended in distilled water, their hydrodynamic size and particle size distributions were determined for the different extract concentrations (Figure 3b). Upon examining the polydispersity indexes (PDIs), it was observed that ZnO-25 and ZnO-100 tend to exhibit more uniform and monodisperse particle sizes (Table 1). In contrast, ZnO-50, with a PDI of 0.51, shows a greater tendency toward polydispersity, aligning with the size distribution displayed in Figure 3b. Notably, ZnO-25 NPs show two separate particle sizes compared to those obtained with 50% and 100%. According to zeta potential values, ZnO NPs derived from *E. crassipes* extract using zinc nitrate as a precursor exhibit electropositive values, with enhanced stability observed for ZnO-25 (Table 1).

**Figure 4:**
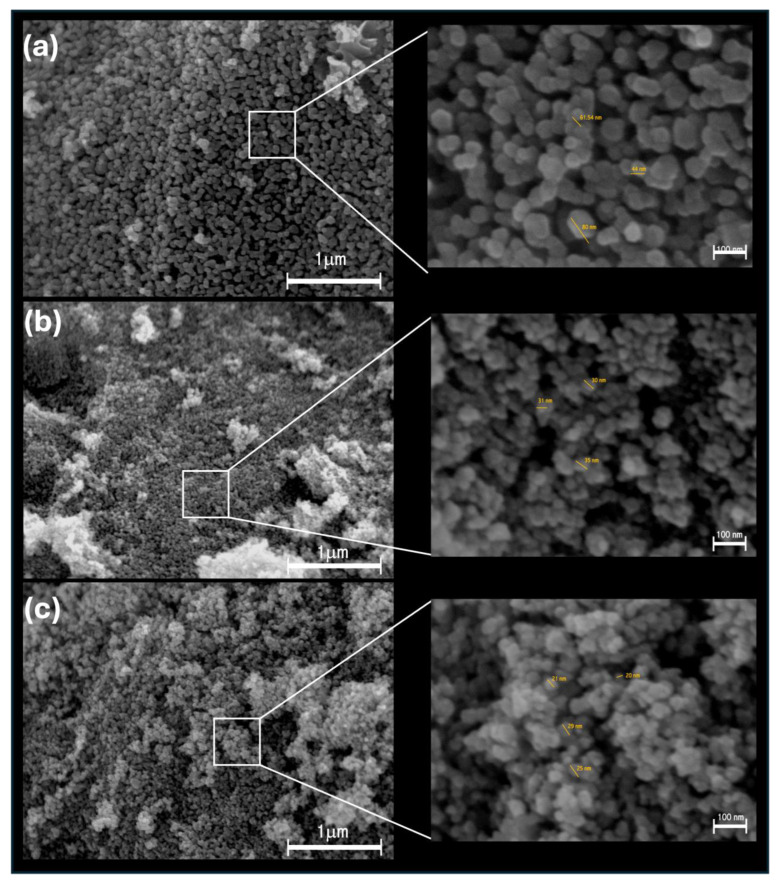
FESEM micrographs of ZnO NPs: (**a**) ZnO-25, (**b**) ZnO-50, and (**c**) ZnO-100. Micrographs on the left at 25,000× and on the right at 100,000× magnification.

FESEM images show different size variations in ZnO NPs depending on the concentration of *E. crassipes* extract. ZnO-25 and ZnO-50 exhibit semi-spherical morphologies typical of chemically synthesized ZnO (Figure 4a–c). Specifically, ZnO-25 sizes range from 50 to 80 nm, while ZnO-50 varies from 40 to 57 nm. In contrast, ZnO-100 displays agglomerated nanoparticles with a less defined morphology, sizes ranging from 20 to 40 nm (see Figure 4 insets b and c). DLS and Electrophoretic Light Scattering (ELS) analyses confirm the tendency of ZnO NPs to agglomerate in water, resulting in relatively larger particle sizes.

The fungal strains isolated from building walls with evident fungal contamination, which exhibit abundant sporulation in culture media and are known in the literature as colonizers of built surfaces, were selected. The strains were identified at the genus level as *Phoma* sp. (H4), *Stachybotrys* sp. (H7), *Fusarium* sp. (H8), and *Aspergillus* sp. (H10). The antifungal activity of ZnO nanomaterials demonstrated significant variations among the different fungal strains and extract concentrations of *E. crassipes* (Table 3). In all instances, the mode of action was fungicidal, with ZnO-25 showing the greatest antifungal effectiveness, as indicated by the lowest MIC and MFC values. An exception was observed with *Aspergillus*, which displayed consistent MFC values regardless of the extract concentration. The most effective activity was against *Phoma* sp. (H4), requiring low MIC/MFC concentrations of ZnO-25 (78 µg/mL) for effective inhibition. In contrast, *Stachybotrys* sp. (H7) exhibited the highest resistance to the action of ZnO NPs.

**Figure 5:**
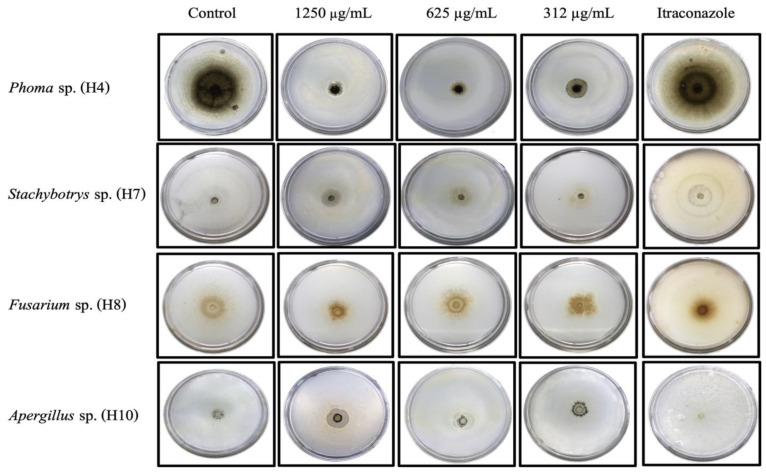
Poisoned plate of ZnO-25 against *Phoma* sp., *Aspergillus* sp., *Fusarium* sp., and *Stachybotrys* sp. Positive control: Itraconazole. Negative control: MEAC medium without any inhibitory compound.

In the poisoned plate assays, ZnO-25 NPs were used because they showed the highest antifungal activity in earlier microdilution tests, applying the three highest MIC/MFC concentrations (Table 3). The results exhibited a typical dose–response effect; higher concentrations of ZnO led to a greater inhibition of fungal radial growth (Figure 5). The analysis of variance (ANOVA) revealed statistically significant differences between the control group (no NPs) and the treatments with different concentrations of ZnO-25, with an F-value of 79.3 (*p* < 0.05). However, among the concentrations of 1250, 625, and 312 µg/mL, no significant differences were found (*p* > 0.05). Contrary to the findings in the microdilution assay, the susceptibility of fungi to ZnO-25 showed that radial inhibition values were higher for *Phoma* sp., followed by *Aspergillus* sp., *Stachybotrys* sp., and lastly *Fusarium* sp., which exhibited the least growth inhibition by the effect of ZnO NPs (Table 4).

## 4. Discussion

The use of plant extracts in the synthesis of ZnO for obtaining and stabilizing agents has been extensively reported [19,20]. The functional groups present in a plant extract can significantly influence the nucleation and growth of NPs, directly affecting the size, morphology, and physical and chemical properties of ZnO. Specifically, the phenolic groups in the extract, which contain OH- groups, facilitate intermolecular interactions that can contribute to the adhesion of organic compounds on the surface of ZnO. This is reflected in the presence of bands in the FTIR spectrum attributed to organic residues even after calcination, as demonstrated by Matinise et al. [21] in the formation of ZnO with *Moringa oleifera* extract, where phytochemicals leave Zn^2+^ metal ions chelated and stabilized during the calcination process. According to previous work [11], the aqueous extract of *E. crassipes* contains polyphenols and flavonoids, which are lightweight volatile compounds. Although the mechanism of extract-mediated synthesis has not been fully understood, it has been assumed that amine and carboxylic groups from the plant extract may adsorb onto the surface of the as-synthesized ZnO particles, as reported with the extract from *Nigella sativa* seeds [22]. These molecules may attach to the surface via hydrogen bonds or electrostatic forces, ensuring strong adherence even after rinsing and drying processes [23]. The thermal decomposition of these organic molecules begins between 60 °C and 80 °C. Consequently, the as-synthesized materials were subjected to a drying process at 70 °C, which likely induced the partial decomposition of the polyphenols and flavonoids on the ZnO surface, resulting in the retention of organic moieties—specifically, the C-H groups from aromatic rings and C-OH groups from phenolic structures [24,25]. Following calcination, these residual groups were further degraded, predominantly leaving behind C-O groups, which may correspond to the band observed at 1736 cm^−1^.

Despite the presence of these organic residues from *E. crassipes*, no adverse effect was observed on the Wurtzite structure of ZnO. However, variations were noted in the peak widths between ZnO-25 and ZnO-100, which can be attributed to a reduction in the crystallite size and changes in the material crystallinity [26,27].

Regarding the crystallite size decrease (Table 1), as the extract concentration increases, it is suggested that the extract plays a crucial role in controlling the growth of NPs. The crystallite sizes reported in the literature for ZnO synthesis assisted by plant extracts vary between 15 and 59 nm and are influenced by the type of precursor, the thermal treatment, and the plant species [10,28,29,30]. Vanathi et al. [9] reported a crystallite size of 32 nm using the same precursor and *E. crassipes* extract, which is larger than the sizes observed in this study with the three different concentrations of the aqueous extract.

Although other studies, such as that of Faisal et al. [31], have reported hydrodynamic sizes of up to 66 nm for ZnO NPs obtained from aqueous fruit extracts of *Myristica fragrans*, our ZnO-25 NPs exhibited larger sizes, reaching 163 nm. No correlation was observed between the presence of the extract and the size distribution, which could be attributed to the agglomeration of ZnO-50 and ZnO-100 NPs, as evidenced in the FESEM micrographs. FESEM images revealed variations in the size of the different ZnO NPs depending on the concentration of *E. crassipes* extract. While individual nanoparticles could be identified, at higher magnification, the presence of small agglomerates was also observable. A well-defined shape was not clearly discernible for any of the nanomaterials; however, for ZnO-25 and ZnO-50, round-shaped morphologies ranging from 50 to 80 nm in size were noticeable (Figure 4a–c). In contrast, for ZnO-100 (Figure 4c), larger agglomerates composed of smaller particles (20–40 nm) were detected. The presence and content of the *E. crassipes* extract did not exert a significant effect on the shape of the particles, as similar morphologies have been reported for ZnO when zinc nitrate is used in combination with other plant extracts [32,33]. It is important to note that nanoparticles exhibit different behaviors depending on the media in which they are applied. This section discusses three different techniques that provide information on the size of the obtained nanoparticles. FESEM is a technique that allows for the high-resolution observation of particle morphology and shape. However, it should be noted that these results reflect the behavior of particles that are perfectly dried and dispersed on carbon tape. This measurement is crucial as it represents the actual size of the particles as prepared. Nonetheless, since the nanoparticles are intended for microbiological applications, understanding how they agglomerate when dispersed in water is essential. Therefore, measuring the hydrodynamic size, which is generally larger than the particle size observed by microscopy, is important. In this study, the hydrodynamic sizes were significantly larger than the sizes observed by microscopy. The Z average for the ZnO-25 nanoparticles was the lowest, also showing a low PDI, indicating lesser polydispersity compared to the other samples. The measured values suggest that small groups of three or four nanoparticles form when dispersed in water, likely due to the presence of surface organic groups that interact through electrostatic forces with water, promoting the formation of small aggregates since the content of organic residuals is minimal for ZnO compared to other samples. The formation of these agglomerates can be confirmed by the low zeta potential values; as a reference, this parameter is used as a criterion for the stability of colloidal suspensions (a zeta potential value greater than ±30 mV is associated with colloidal stability). From the zeta potential results, it can be seen that the samples ZnO-50 and ZnO-100 exhibit values close to zero, indicating poor stability and a tendency to sediment. This is also evident from the FESEM images. The high polydispersity of these two materials also contributes to the rapid sedimentation of the nanoparticle agglomerates, as shown by the width of the corresponding distribution curves (Figure 3b). These results allow us to select the ZnO-25 nanomaterial as the best candidate for biological testing because lower sedimentation and agglomeration can be expected, which could improve the nanoparticles’ diffusion through biological media.

For nanomaterials, the presence of nanocrystals is also considered an important contribution to their final properties in specific applications. It has been reported that the crystallite size influences, for instance, the photocatalytic activity of ZnO nanoparticles [34]. From the calculations made for the three nanomaterials, no significant changes were observed in the crystallite size of ZnO-50 and ZnO-100 due to the different concentrations of the extract. However, ZnO-25 exhibited the smallest calculated size, being around 11 nm. This suggests that the nanoparticles observed by FESEM could be composed of a few crystallites along with some amorphous material. Thus, the lower the concentration of the plant extract, the smaller the particles and crystallites formed. Zeta potential analyses for NPs synthesized with different concentrations of the extract and using zinc nitrate (Zn(NO_3_)_2_·6H_2_O) as a precursor showed that ZnO-25 tends towards greater stability with a zeta potential of 17.4 mV, higher than the values observed for ZnO-50 and ZnO-100, which do not exhibit colloidal stability.

Additionally, the absorption edges near 370 nm correspond to the optical absorbance of the wurtzite crystal structure of ZnO [35]. This band is specifically associated with the intrinsic absorption of electronic transitions of O2p → Zn3d, from the valence band to the conduction band [36]. When prepared without plant extract, ZnO materials are generally white-in-color powders. The use of the plant extract, which exhibits a brown translucent color when concentrated, provides a light coloring to the ZnO dried samples. As expected, as the plant extract was diluted, the brownish color shifted to a pale yellow. The three ZnO samples prepared with the extract exhibited a pale green-yellow hue, varying in intensity according to the dilution of the extract; ZnO-100 had the most intense color, while ZnO-25 showed the least intensity. When the ZnO powders were calcined, the coloring for the three samples shifted to pale yellow, decreasing in intensity as the concentration of the extract decreased, with ZnO-25 exhibiting a white-yellowish color. Despite the slight coloring of the samples, the DR-UV-Vis spectra (Figure S2) did not show significant changes in either the absorption edge or the Eg values compared to the literature [12]. The calculated Eg values ranged from 3.22 to 3.27 eV, corresponding to 379 to 385 nm, indicating the absorption of UV-A light, which is very close to the visible region of the spectrum. This finding is consistent with previous XRD analyses, indicating that neither the *E. crassipes* extract nor its dilutions significantly affect the optical properties of ZnO NPs.

Adsorption–desorption N_2_ isotherms show that, at low relative pressures, the three concentrations of the ZnO extract result in the adsorption of relatively low volumes, with values exceeding 200 cm^3^/g at a relative pressure of 0.98. This indicates an increase in the surface area of ZnO proportional to the concentration of the extract. In Figure 2a, narrow hysteresis loops are observed at relative pressures close to 0.9, although the limited amount of data in the graph prevents definitive confirmation. Therefore, the isotherm is classified as type II according to IUPAC [37].

The specific BET surface area of chemically synthesized ZnO NPs from zinc salts without additives is reported at 10 m^2^/g [30], significantly lower than observed in this study. This suggests that the organic molecules in the extract influence the porosity of the solid and the adsorption energy between the adsorbent and the adsorbate, favoring an increase in the surface area of ZnO. According to Zhang et al. [38], for the effective modification of the ZnO surface, the BET area should exceed the range of 1 to 15 cm^2^/g. The BET constant, associated with the heat of adsorption and adsorbent–adsorbate interactions, is defined as the net molar energy of adsorption. Generally, low values (C < 20) suggest that the interactions between the NP surface and the adsorbate are relatively strong. There is a direct relationship between the concentration of the extract and the value of C. According to the IUPAC classification, the reported values for average pore size (Table 2) fall within the range of mesopores; however, the broad distribution of pore size (approximately 10 to 100 nm), with a low volume of gas adsorbed, indicates the presence of macropores in the sample (Figure 2b). Additionally, fractal dimension coefficients between 2.2 and 2.3 are reported, implying a relatively smooth surface (Table 2). Since the variations are minimal, it is inferred that there is no direct correlation between the dilution of the extract and the roughness of the ZnO surface, a critical factor in its interaction with biological systems and its potential antifungal activity.

The strains isolated from environments affected by SBS include *Aspergillus* sp., *Stachybotrys* sp., *Fusarium* sp., and *Phoma* sp. These fungi are pertinent to the present study due to their ability to produce mycotoxins and cause respiratory diseases that compromise human health and exhibit resistance to conventional treatments [4,39,40,41].

The antifungal activity of ZnO nanoparticles synthesized from *E. crassipes* extract is demonstrated by their varying degrees of inhibitory concentrations against fungal strains isolated from building materials. ZnO-25 exhibits potent fungicidal effects at concentrations as low as 78 µg/mL for *Phoma* sp. and up to 1250 µg/mL for *Stachybotrys* sp. In contrast, higher concentrations are required for ZnO-50 and ZnO-100 to achieve similar inhibitory effects, which correlates with their higher polydispersity index (PDI) and larger crystallite sizes. The superior antifungal performance of ZnO-25 is likely due to electrostatic interactions between the positively charged ZnO surface (zeta potential) and negatively charged fungal cell membranes, which consist of phospholipids and proteins. This interaction facilitates the adherence of ZnO to fungal cells, enhancing its fungicidal activity, like the observed mechanisms in antibacterial applications on ZnO [42]. This can be compared with previous studies, such as those by Soria-Castro et al. [16], who reported that ZnO nanoparticles synthesized via the sol–gel method affected two fungal strains including *Aspergillus* sp., with an MIC of 39 µg/mL requiring a higher concentration (1250 µg/mL) to observe fungicidal effects.

The poisoned plate assay revealed that *Phoma* sp. exhibited high inhibition percentages, whereas *Fusarium* sp. showed no significant growth differences at concentrations of 624 and 312 µg/mL (Figure 5). Trzcińska-Wencel et al. [43] also studied ZnO nanoparticles biosynthesized from fungal extracts against various phytopathogens, including strains of genera *Fusarium* and *Phoma*. Using the poisoned plate method, they found that most *Fusarium* strains were not inhibited by the nanoparticles, while *Phoma* showed a 60% inhibition rate at 1000 µg/mL, similar to the obtained results in the present work. This could be due to Fusarium strains being multicellular with 3–4 septa per cell, requiring higher amounts of nanoparticles to inhibit each septum, making them more resistant to the effects of nanoparticles compared to septa-free *Phoma* conidia [44].

Understanding the precise mechanisms of ZnO NPs’ antimicrobial action remains a subject of research. Three main pathways have been described [45]: physical interactions involve electrostatic effects where ZnO NPs approach and disrupt the conidium membranes, allowing for the intermembrane component to release, corroborating some of the obtained MFC results. Chemically, upon contact with the microorganism, ZnO releases Zn^2+^ cations and generates reactive oxygen species (ROS) causing oxidative stress [46], leading to cellular damage observed as reduced growth in the poisoned plate assay. There are several reports on the mechanisms of the biological activity of ZnO nanomaterials, with two widely accepted and demonstrated mechanisms. The first involves the generation of reactive oxygen species (ROS), which are responsible for causing oxidative stress in cells [38]. As a photoactive semiconductor that absorbs UV-A light, ZnO can generate electron–hole pairs when exposed to solar radiation, leading to the production of hydroxyl radicals that interact with biomolecules [12]. The second mechanism concerns the release of zinc cations [47,48]. In the presence of certain biomolecules or biological media, Zn^2+^ ions may be released from the ZnO network [49]. However, the results regarding the role and ion release capacity of ZnO are controversial, as these depend on the media and the type of cells involved [50]. Therefore, we suggest that the possible mechanism by which fungi are being inhibited could involve the presence of some oxidant species generated by ZnO, but additional experiments are needed to confirm this. Given these outcomes, ZnO-25 appears to be a promising antifungal candidate for evaluation as a cement compound additive in the construction industry.

## 5. Conclusions

This study demonstrates that the organic compounds extracted from *E. crassipes* using water, when used as a reaction medium, facilitate the synthesis of ZnO nanoparticles using zinc nitrate. Although the impact of the extract concentration on the shape of the nanoparticles was not definitive, it appears that the organic compounds may guide the formation of small, round-shaped nanoparticles. Variations in the concentration of the extract induce subtle yet significant alterations in some properties of ZnO. It can be assumed that the presence of small amounts of organic groups attached to the surface of ZnO significantly enhances its bioactivity compared to higher concentrations (ZnO-50 and ZnO-100). The more diluted extract leads to less agglomeration of nanoparticles and, together with a higher electropositive zeta potential, improves dispersion in water. This enhancement in dispersion facilitates greater contact with fungi, as indicated by the low values for the MIC and MFC. The observed bioactivity may be attributed to an electropositive and less rough nanoparticle surface. Since the C constant from the BET equation indicates interactions between the surface and adsorbed molecules, it is reasonable to infer that the extract content significantly modifies the surface properties of the synthesized nanomaterials. This modification decreases the surface’s interaction with other molecules as the extract concentration is reduced, as observed with ZnO-25, suggesting lower porosity and reduced adsorption heat. This may result in a balance allowing for easy detachment between nanoparticles and biomolecules from the fungal membrane while still causing damage, though further experiments are required to confirm this hypothesis. Regarding the antifungal activity against strains associated with SBS, the results for ZnO with all extracts suggest a fungicidal rather than fungistatic mechanism of action, enhancing the competitiveness of these nanoparticles compared to other ZnO materials synthesized via bio-assisted methods. The poisoned plate assay reveals that ZnO-25 exhibited the most significant radial inhibition against *Phoma* sp., followed by *Aspergillus* sp., *Stachybotrys* sp., and *Fusarium* sp., with the latter being the least affected. Given its efficacy against these strains, ZnO-25 emerges as a promising antifungal agent for incorporation into compounds used in the construction sector.

## Figures and Tables

**Table 1:** Physical and chemical parameters of ZnO NPs synthesized via a bio-assisted method using *E. crassipes* extract at different concentrations.

Sample	Crystallite Size (nm)	Eg (eV)	Z_avg_ (nm)	PDI	Zeta Potential (mV)
ZnO-25	20.03	3.26	163	0.23	17.40
ZnO-50	17.79	3.22	3406	0.51	3.52
ZnO-100	11.80	3.27	1542	0.38	1.40

**Table 2:** Textural parameters from the adsorption–desorption isotherms of ZnO NPs.

Sample	Surface Area m^2^/g	C	R	D_FHH (des)_	Pore Width (nm)
ZnO-25	26	70.71	0.99	2.27	33
ZnO-50	26	264.13	0.99	2.34	32
ZnO-100	36	172.48	1.00	2.35	32

D_FHH (des)_: fractal dimension coefficient obtained by the Frenkel–Halsey–Hill method from desorption data. C is the BET equation constant of the related adsorbate–adsorbent interaction.

**Table 3:** MIC and MFC values of ZnO NPs obtained at different concentrations of *E. crassipes* extract against isolated fungal strains.

Strain	ZnO-100		ZnO-50		ZnO-25	
	MFC/MIC (μg/mL)	MA	MFC/MIC (μg/mL)	MA	MFC/MIC (μg/mL)	MA
*Phoma* sp. (H4)	312/156	FC	312/156	FC	78/78	FC
*Stachybotrys* sp. (H7)	2500/1250	FC	5000/2500	FC	1250/1250	FC
*Fusarium* sp. (H8)	625/625	FC	1250/625	FC	625/312	FC
*Aspergillus* sp. (H10)	625/312	FC	625/312	FC	625/625	FC

MIC: minimum inhibitory concentration, MFC: Minimum Fungicidal Concentration, MA: mode of action, FC: fungicidal, FG: fungistatic.

**Table 4:** Mycelial growth inhibition (MGI) [%] of ZnO-25 nanoparticles.

Strain	Concentration of ZnO-25 (μg/mL)			Itraconazole (mg/mL)
	1250	625	312	6.6
*Phoma* sp. (H4)	84.1 ± 1.32	80.8 ± 0.57	72.9 ± 5.19	12 ± 0
*Stachybotrys* sp. (H7)	77.1 ± 0.57	70.0 ± 1.73	65.6 ± 3.12	47 ± 4.04
*Fusarium* sp. (H8)	64.5 ± 4.75	53.9 ± 1.89	54.6 ± 3.18	14 ± 0
*Aspergillus* sp. (H10)	74.5 ± 0.76	68.7 ± 4.59	64.5 ± 0.28	0

## Data Availability

On inquiry, the data presented in this study are available from the authors.

## Supplementary Materials

The following supporting information can be downloaded at https://www.mdpi.com/article/10.3390/nano14121007/s1, Figure S1: Kubelka–Munk plot for ZnO nanoparticles (NPs) synthesized using *Eichhornia crassipes* extracts at three different concentrations, as derived from DR-UV-Vis spectra: (a) ZnO-25; (b) ZnO-50; and (c) ZnO-100. Figure S2: DR-UV-Vis spectra of ZnO NPs synthesized from three different concentrations of *E. crassipes* extract.
