# Supplementary material for: Antifungal Activity of ZnO Nanoparticles Synthesized from Eichhornia crassipes Extract for Construction Applications
Source: Nanomaterials (Basel). 2024 Jun 11;14(12):1007. doi: 10.3390/nano14121007 (PMC11206371; doi:10.3390/nano14121007)
Supplement: Supplementary file 1 [file nanomaterials-14-01007-s001.zip › nanomaterials-3034813-supplementary.pdf]

Supplementary material

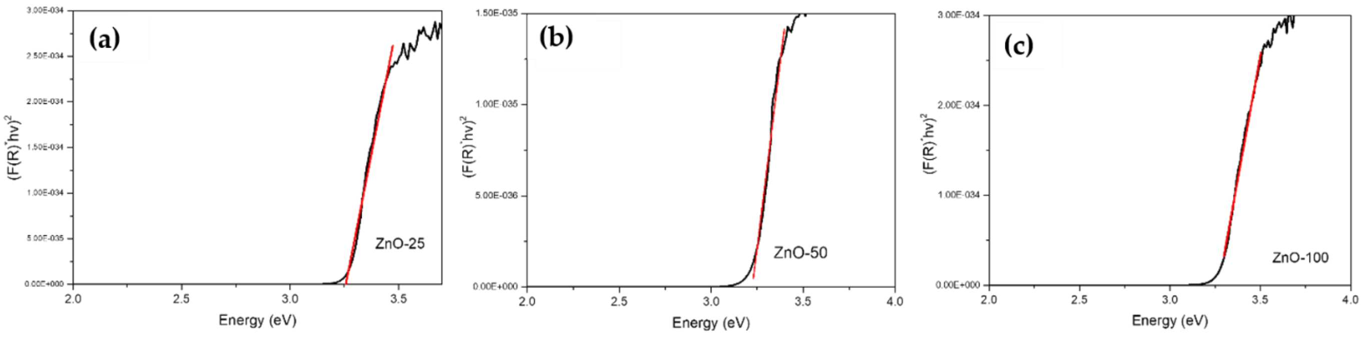

Figure S1. Kubelka-Munk plot for ZnO nanoparticles (NPs) synthesized using *Eichhornia crassipes* extracts at three different concentrations, as derived from DR-UV-Vis spectra: (a) ZnO-25; (b) ZnO-50; (c) ZnO-100.

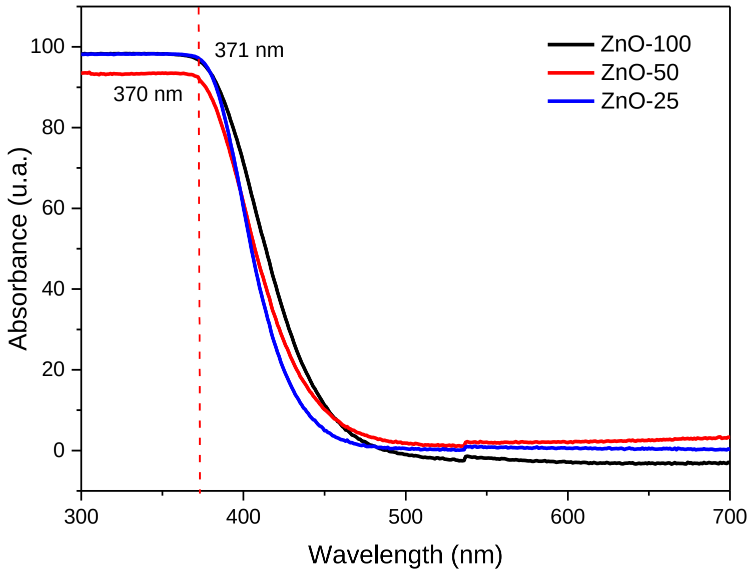

Figure S2. DR-UV-Vis spectra of ZnO NPs synthesized from three different concentrations of E. crassipes extract.
